# Variabilidade da cultura de segurança do paciente em hospitais brasileiros: influência dos fatores contextuais

**DOI:** 10.1590/0102-311XPT052025

**Published:** 2026-03-09

**Authors:** Zenewton André da Silva Gama, Magda Machado de Miranda Costa, Alessandra Anneliese da Silva Souza Medeiros, Cecília Olívia Paraguai de Oliveira Saraiva, Heiko Thereza Santana, Ana Clara Ribeiro Bello dos Santos, Natália Gentil Linhares, Ricardo Alexsandro de Medeiros Valentim, Jailton Carlos de Paiva, Marise Reis de Freitas

**Affiliations:** 1 Departamento de Saúde Coletiva, Universidade Federal do Rio Grande do Norte, Natal, Brasil.; 2 Grupo de Pesquisa Qualidade em Serviços de Saúde, Universidade Federal do Rio Grande do Norte, Natal, Brasil.; 3 Gerência de Vigilância e Monitoramento de Serviços de Saúde, Agência Nacional de Vigilância Sanitária, Brasília, Brasil.; 4 Residência Multiprofissional em Saúde Coletiva, Universidade de Pernambuco, Recife, Brasil.; 5 Departamento de Engenharia Biomédica, Universidade Federal do Rio Grande do Norte, Natal, Brasil.; 6 Laboratório de Inovação Tecnológica em Saúde, Universidade Federal do Rio Grande do Norte, Natal, Brasil.; 7 Instituto Federal do Rio Grande do Norte, Natal, Brasil.; 8 Departamento de Infectologia, Universidade Federal do Rio Grande do Norte, Natal, Brasil.

**Keywords:** Qualidade da Assistência à Saúde, Segurança do Paciente, Cultura Organizacional, Quality of Health Care, Patient Safety, Organizational Culture, Calidad de la Atención de Salud, Seguridad del Paciente, Cultura Organizacional

## Abstract

A segurança do paciente é um desafio estratégico para o Sistema Único de Saúde (SUS) e uma prioridade para a qualidade hospitalar no Brasil. Este estudo descreve a variação da cultura de segurança entre hospitais brasileiros e analisa se fatores estruturais e organizacionais podem explicar parte dessas diferenças. Foi realizado um estudo transversal com dados coletados em 2021 de 42.038 profissionais de saúde que atuam em 304 hospitais públicos e privados, distribuídos por todas as regiões do país. A cultura de segurança foi mensurada a partir da proporção de respostas positivas a 42 afirmações do *Hospital Survey on Patient Safety Culture*, um questionário internacional desenvolvido pela Agência para Pesquisa e Qualidade em Saúde dos Estados Unidos (AHRQ) e adaptado para o contexto brasileiro. As variáveis analisadas incluíram localização geográfica, tipo de gestão e porte hospitalar, por meio de regressão linear multivariada com reamostragem (*bootstrap*). Observou-se ampla variação entre os hospitais nas 12 dimensões avaliadas da cultura de segurança. Duas dimensões foram consideradas como pontos fortes (com médias superiores a 75% de respostas positivas), enquanto uma foi identificada como fragilidade (com média inferior a 50%). O modelo estatístico explicou 37% da variação e apontou como principais fatores associados a localização na Região Nordeste, a gerência pública indireta, o pequeno porte institucional e o *status* de hospital de ensino. Conclui-se que a cultura de segurança do paciente varia significativamente entre hospitais do país e está relacionada a características estruturais que podem ser alvo de políticas públicas e estratégias de gestão. Os achados fortalecem iniciativas como o Programa Nacional de Segurança do Paciente e oferecem subsídios práticos para a tomada de decisão no âmbito do SUS.

## Abstract (en)

Patient safety is a strategic challenge for the Brazilian Unified National Health System (SUS) and a priority for hospital quality. This study describes the variation in safety culture among Brazilian hospitals and analyzes whether structural and organizational factors may explain part of these differences. A cross-sectional study was conducted with data collected in 2021 from 42,038 healthcare providers working at 304 public and private hospitals distributed throughout all regions of the country. Safety culture was measured based on the proportion of positive answers to 42 statements of the *Hospital Survey on Patient Safety Culture*, which is an international questionnaire developed by the U.S. Agency for Health Research and Quality and adapted to the Brazilian context. The variables of interest were geographic location, type of management, and hospital size, which were analyzed using multivariate linear regression with resampling (bootstrap). Considerable variation was found among hospitals for the 12 dimensions of safety culture analyzed. Two dimensions were considered strengths (average of more than 75% positive responses), while one was identified as a weakness (average of less than 50%). The statistical model explained 37% of the variation. The main associated factors were location in the Northeast region, indirect public management, small institutional size, and teaching-hospital status. In conclusion, patient safety culture varies significantly among hospitals in the country and is related to structural characteristics that could be the target of public policies and management strategies. The present findings strengthen initiatives such as the Brazilian National Patient Safety Program and offer practical support for decision-making within the SUS.

## Abstract (es)

La seguridad del paciente es un reto estratégico para el Sistema Único de Salud (SUS) y una prioridad para la calidad hospitalaria en Brasil. Este estudio describe la variación de la cultura de seguridad entre los hospitales brasileños y analiza si los factores estructurales y organizativos pueden explicar parte de estas diferencias. Se realizó un estudio transversal con datos recopilados en 2021 de 42.038 profesionales sanitarios que trabajan en 304 hospitales públicos y privados, distribuidos por todas las regiones del país. La cultura de la seguridad se midió a partir de la proporción de respuestas positivas a 42 afirmaciones de la *Hospital Survey on Patient Safety Culture*, un cuestionario internacional desarrollado por la Agencia para la Investigación y la Calidad en la Salud de los Estados Unidos (AHRQ) y adaptado al contexto brasileño. Las variables analizadas incluyeron la ubicación geográfica, el tipo de gestión y el tamaño del hospital, mediante regresión lineal multivariante con remuestreo (*bootstrap*). Se observó una amplia variación entre los hospitales en las 12 dimensiones evaluadas de la cultura de seguridad. Dos dimensiones se consideraron puntos fuertes (con una media superior al 75% de respuestas positivas), mientras que una se identificó como punto débil (con una media inferior al 50%). El modelo estadístico explicó el 37% de la variación y señaló como principales factores asociados la ubicación en la región Nordeste, la gestión pública indirecta, el pequeño tamaño institucional y la categoría de hospital escuela. Se concluye que la cultura de seguridad del paciente varía significativamente entre los hospitales del país y está relacionada con características estructurales que pueden ser objeto de políticas públicas y estrategias de gestión. Los hallazgos refuerzan iniciativas como el Programa Nacional de Seguridad del Paciente y ofrecen subsidios prácticos para la toma de decisiones en el ámbito del SUS.

## Introdução

A segurança do paciente tem se consolidado como uma prioridade global e um indicador essencial da qualidade nos serviços de saúde ^1^. No Brasil, fortalecer a cultura de segurança do paciente tornou-se uma diretriz estratégica do Sistema Único de Saúde (SUS), incorporada ao Programa Nacional de Segurança do Paciente (PNSP) ^2^. Essa responsabilidade é compartilhada com os serviços de saúde por meio dos Núcleos de Segurança do Paciente (NSP), instituídos como estruturas de governança clínica voltadas à promoção de práticas seguras ^3^.

A cultura de segurança do paciente envolve valores, atitudes, competências e comportamentos organizacionais que favorecem o comprometimento de uma organização com a segurança assistencial, substituindo a lógica da culpa e punição por um ambiente de aprendizagem contínua ^2^. Trata-se de um conceito complexo, ainda em evolução ^4^, que vem sendo operacionalizado a partir de múltiplas dimensões, como trabalho em equipe, comunicação aberta, apoio da gestão, aprendizagem organizacional e resposta não punitiva a erros ^5^.

Promover essa cultura exige mudanças estruturais e relacionais. Diretrizes internacionais e o próprio PNSP recomendam: (i) a criação de estruturas de liderança, como os NSP; (ii) o monitoramento regular da cultura com *feedback* e intervenções de melhoria; (iii) a capacitação interprofissional para o trabalho em equipe e práticas seguras; e (iv) a adoção de processos sistemáticos para identificar e mitigar riscos ^1^ ^,^ ^2^ ^,^ ^3^ ^,^ ^6^.

A Avaliação Nacional da Cultura de Segurança do Paciente, realizada pela Agência Nacional de Vigilância Sanitária (Anvisa), em 2021, permitiu que mais de 300 hospitais brasileiros monitorassem suas práticas com base em evidências ^7^, estimulando a autorregulação e subsidiando decisões de gestão clínica e institucional ^8^. Esses dados são estratégicos também para políticas públicas, permitindo identificar hospitais risco ou com boas práticas (*benchmarks*) ^9^.

A Organização Mundial da Saúde (OMS) reconhece o monitoramento da cultura de segurança como um componente central dos sistemas nacionais de segurança do paciente, recomendando sua adoção como um dos indicadores globais de progresso no Plano de Ação Global para a Segurança do Paciente 2021-2030 ^1^. Em alinhamento com essa diretriz, o Brasil incorporou esse indicador ao Plano Integrado para a Gestão Sanitária da Segurança do Paciente 2021-2025, que estabelece como meta a participação de, no mínimo, 40% dos hospitais com leitos de unidade de terapia intensiva (UTI) nas avaliações nacionais da cultura de segurança até 2025 ^10^.

O objetivo deste estudo é descrever a variabilidade da cultura de segurança do paciente em hospitais brasileiros e analisar se características contextuais podem prever essa variação.

Evidências internacionais apontam que entender onde e como as intervenções ocorrem é tão importante quanto seu conteúdo ^11^. Embora estudos associem à cultura de segurança do paciente desfechos clínicos ^12^, ainda são raras as análises nacionais com abordagem multivariada. Esta pesquisa busca preencher essa lacuna, contribuindo com a ciência da implementação e subsidiando políticas públicas mais sensíveis às realidades institucionais do SUS.

## Métodos

### Desenho do estudo

Trata-se de um estudo transversal com análise secundária de dados provenientes da Avaliação Nacional de Cultura de Segurança do Paciente em Hospitais realizada em 2021, sob coordenação da Anvisa, em parceria com a Universidade Federal do Rio Grande do Norte (UFRN). Os dados foram obtidos por meio do sistema eletrônico E-Questionário de Cultura de Segurança Hospitalar (disponível em: https://csp.qualisaude.telessaude.ufrn.br/) e complementados com variáveis do Cadastro Nacional de Estabelecimentos de Saúde (CNES), mantido pelo Departamento de Informática do SUS (DATASUS).

### População de estudo e critérios de exclusão

Foram incluídos todos os hospitais brasileiros com pelo menos 20 leitos que participaram da avaliação nacional. Os critérios de exclusão foram: (a) menos de 10 questionários válidos por hospital; (b) inconsistência entre código CNES e nome do hospital (n = 2); (c) hospitais dia ou isolados (n = 1); e (d) valores atípicos no índice de cultura (> 3 vezes o intervalo interquartil) (n = 4).

**Figura 1:**
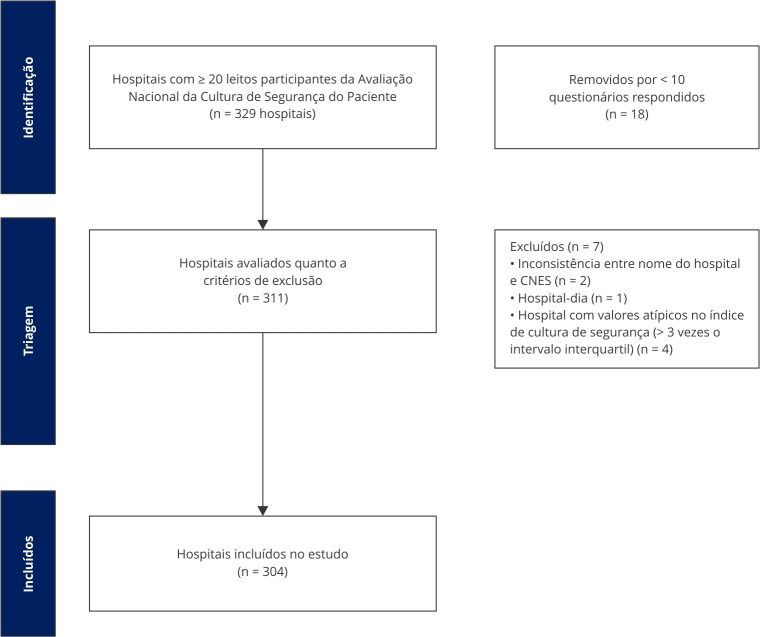
Etapas de seleção dos hospitais participantes no estudo.

O processo de elegibilidade e exclusão está descrito em fluxograma apresentado na Figura 1.

CNES: Cadastro Nacional de Estabelecimentos de Saúde. Fonte: dados do estudo, 2025.

### Instrumento de medida da cultura de segurança

Foi utilizada uma versão brasileira, traduzida, adaptada e validada do instrumento *Hospital Survey on Patient Safety Culture* (HSOPS; Pesquisa Hospitalar sobre a Cultura de Segurança do Paciente, desenvolvido pela Agência para Pesquisa e Qualidade em Saúde dos Estados Unidos - AHRQ) ^13^. Trata-se de um questionário internacional padronizado, que avalia as percepções das equipes hospitalares sobre segurança do paciente, erros e notificações. Ele contém 42 perguntas organizadas em 12 dimensões, com escalas de 5 pontos para frequência (“Nunca” a “Sempre”) ou concordância (“Discordo fortemente” a “Concordo fortemente”).

As 12 dimensões avaliadas são:

1. Frequência de notificação de incidentes (3 itens);

2. Percepção de segurança (4 itens);

3. Expectativas e ações da direção/supervisão da unidade/serviço que favorecem a segurança (4 itens);

4. Aprendizagem organizacional/melhoria contínua (3 itens);

5. Trabalho em equipe na unidade/serviço (4 itens);

6. Abertura para comunicação (3 itens);

7. *Feedback* e comunicação sobre erros (3 itens);

8. Resposta não punitiva aos erros (3 itens);

9. Dimensionamento de pessoal (4 itens);

10. Apoio da gerência à segurança (3 itens);

11. Trabalho em equipe entre unidades (4 itens);

12. Mudanças de turno e transições entre unidades/serviços (4 itens).

### Variáveis do estudo

A variável dependente foi o Índice de Cultura de Segurança do Paciente (ICSP), calculado pela média da porcentagem de respostas positivas nos 42 itens do questionário HSOPS.

(1) $$ICSP=\frac{x_{1}+x_{2}+x_{3}\ldots +x_{42}}{42}$$

onde *x* corresponde ao percentual de resposta positiva de cada item do questionário.

As variáveis independentes foram: região geográfica (Norte, Nordeste, Sul, Sudeste, Centro-oeste); natureza jurídica (administração pública, privada filantrópica, privada empresarial); tipo de hospital (geral, especializado); porte (pequeno: até 50 leitos; médio: 51-150; grande: ≥ 151); atendimento ao SUS (sim, não); percentual de leitos SUS (0%; 1-49%; 50-99%; 100%); hospital de ensino (sim, não); tipo de gerência (pública direta, pública indireta, privada sem fins lucrativos, privada empresarial); presença de leitos de UTI, cirúrgicos e de COVID-19 (não, sim).

### Tratamento de dados ausentes

Foram aplicadas análises por pares completos. Não foi realizada imputação de dados. As proporções de respostas ausentes foram baixas (< 1%) e não comprometeram a robustez das análises. Hospitais com preenchimento incompleto em variáveis críticas foram excluídos conforme critérios já descritos.

### Coleta e análise de dados

As informações foram exportadas do sistema do E-Questionário de Cultura de Segurança Hospitalar em formato CSV. As variáveis contextuais foram extraídas do CNES.

Para evitar multicolinearidade, a variável “natureza jurídica” foi dicotomizada em hospitais públicos (sim/não), e a variável “gerência” foi mantida categórica (quatro níveis).

Variáveis numéricas como número de leitos de UTI, cirúrgicos e de COVID-19 foram transformadas em variáveis binárias (0, ≥ 1).

A caracterização da amostra foi feita com frequências absolutas e relativas. A distribuição do ICSP foi explorada com *boxplots* e os pontos de corte adotados foram: média > 75% para dimensões fortalecidas; < 50% para fragilidades.

Após teste de Shapiro-Wilk (p = 0,001), utilizamos o teste U de Mann-Whitney para comparações entre dois grupos e Kruskal-Wallis para três ou mais, com *post hoc* por comparações par a par (*pairwise*).

As variáveis com associação estatística ao ICSP (p < 0,05) foram inseridas em modelo de regressão linear múltipla com variáveis *dummy*. O modelo atendeu aos pressupostos de linearidade, homoscedasticidade, independência dos resíduos (Durbin-Watson = 1,828), ausência de multicolinearidade e distribuição dos resíduos.

Empregou-se a técnica de reamostragem *bootstrap* (2.000 amostras; intervalo de 95% de confiança com correção *bootstrap* do tipo corrigido e acelerado pelo viés - IC95% BCa) para garantir estimativas robustas ^14^. As análises foram conduzidas no software IBM SPSS Statistics, versão 29.0 (https://www.ibm.com/), com nível de significância de 5%.

### Aspectos éticos

Este estudo utilizou dados secundários autorizados pela Anvisa e aprovados pelo Comitê de Ética em Pesquisa do Hospital Universitário Onofre Lopes (parecer nº 5.501.454). Nenhuma informação identificável de pacientes, profissionais ou hospitais foi utilizada.

## Resultados

### Caracterização da amostra

Foram analisados dados de 304 hospitais e 42.038 profissionais nas análises descritiva e bivariada, e de 302 hospitais e 42.014 profissionais na regressão multivariada. Todas as cinco regiões brasileiras estiveram representadas, com destaque para a Região Nordeste (46,7%). Apenas o Acre não teve hospitais incluídos.

A média de respondentes por hospital foi de 138,2 (intervalo: 10 a 1.140), com percentual médio de resposta de 62,6% (intervalo: 1,7% a 100%). A maioria dos hospitais era geral (84,5%), pública (55,3%), de grande porte (47,7%), com gerência pública direta (30,3%), não de ensino (57,9%) e prestadora de serviços ao SUS (83,6%). Cerca de 90% dispunham de leitos de UTI e cirúrgicos, enquanto aproximadamente metade tinha leitos habilitados para COVID-19. A Tabela 1 apresenta o detalhamento das características institucionais.

**Tabela 1:** Caracterização dos hospitais participantes (n = 304), segundo localização, porte, natureza jurídica e relação com o Sistema Único de Saúde (SUS).

Características	n	%
Região		
Norte	22	7,2
Nordeste	142	46,7
Centro-oeste	25	8,2
Sudeste	71	23,4
Sul	44	14,5
Tipo de estabelecimento		
Hospital geral	257	84,5
Hospital especializado	47	15,5
Porte do hospital		
Pequeno (até 50 leitos)	26	8,6
Médio (51-150 leitos)	133	43,8
Grande (> 150 leitos)	145	47,7
Leitos de UTI		
Não	37	12,2
Sim	267	87,8
Leitos cirúrgicos		
Não	22	7,2
Sim	282	92,8
Leitos de COVID-19		
Não	143	47,0
Sim	161	53,0
Hospital de ensino		
Não	176	57,9
Sim	128	42,1
Hospital público		
Não	136	44,7
Sim	168	55,3
Natureza jurídica		
Administração pública	168	55,3
Entidade empresarial	56	18,4
Entidade filantrópica	80	26,3
Gerência		
Pública direta *	100	32,8
Pública indireta **	73	23,9
Privada filantrópica	66	21,6
Privada empresarial	66	21,6
Atende ao SUS		
Não	50	16,4
Sim	254	83,6
Leitos do SUS (%)		
0	50	16,5
1-49	19	6,3
50-99	137	45,2
100	97	32,0
Público de fato (> 50% leitos do SUS) ***		
Não	69	22,8
Sim	234	77,2

SUS: Sistema Único de Saúde; UTI: unidade de terapia intensiva.

* Inclui hospitais sob gestão direta de órgãos públicos;

** Refere-se à gestão por entidades públicas autônomas (p.ex.: empresas públicas ou fundações);

*** Refere-se a hospitais com ≥ 50% dos leitos destinados ao SUS.

### Variabilidade na cultura de segurança do paciente

**Figura 2:**
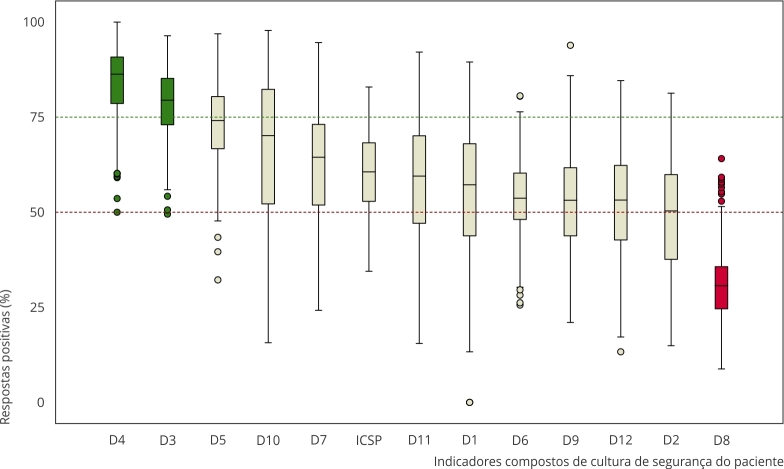
Distribuição do Índice de Cultura de Segurança do Paciente (ICSP) e das 12 dimensões avaliadas pelo instrumento *Hospital Survey on Patient Safety Culture* (HSOPS) em hospitais brasileiros (n = 304).

A Figura 2 mostra a distribuição do ICSP e das 12 dimensões avaliadas pelo instrumento HSOPS. Os *boxplots* evidenciam ampla variabilidade entre os hospitais, com dispersão mais acentuada na dimensão “Apoio da gerência à segurança” (dimensão 10). A maioria das dimensões apresentou medianas entre 50% e 75%, sendo que duas dimensões foram consideradas fortalezas (dimensões 3 e 4, com porcentagem de respostas positivas - PRP - superior a 75%) e uma foi classificada como fragilidade (dimensão 8, com PRP < 50%).

Nota: as cores dos *boxplots* indicam o desempenho por dimensão: verde: ≥ 75% (fortalezas); bege: 50%-74% (moderado); vermelho: < 50% (fragilidade). Linhas tracejadas verde (75%) e vermelha (50%) como referências analíticas. D1: Frequência de notificação de incidentes; D2: Percepção de segurança; D3: Expectativas e ações da direção/supervisão da unidade/serviço que favorecem a segurança; D4: Aprendizagem organizacional/melhoria contínua; D5: Trabalho em equipe na unidade/serviço; D6: Abertura para comunicação; D7: *Feedback* e comunicação sobre erros; D8: Resposta não punitiva aos erros; D9: Dimensionamento de pessoal; D10: Apoio da gerência à segurança; D11: Trabalho em equipe entre unidades; D12: Mudanças de turno e transições entre unidades/serviços.

### Associação entre fatores contextuais e o ICSP

**Figura 3:**
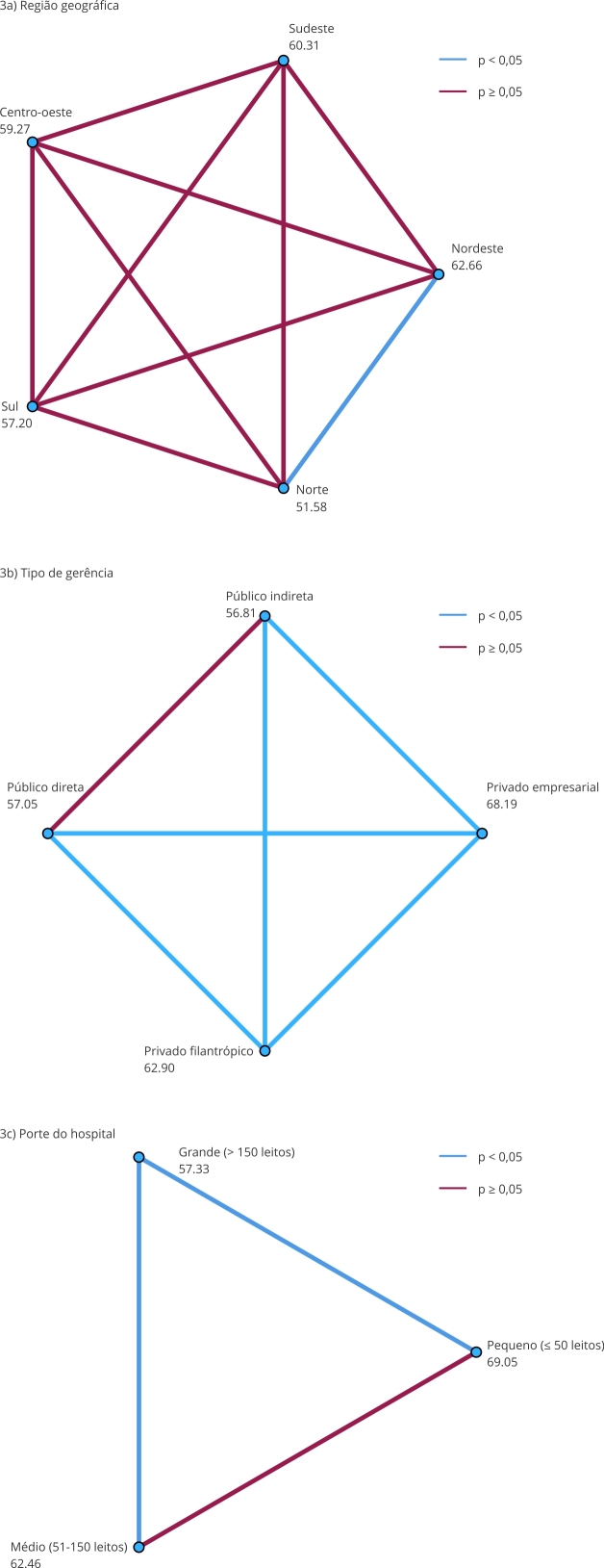
Comparações *post hoc* entre categorias de variáveis politômicas associadas ao Índice de Cultura de Segurança do Paciente (ICSP), segundo teste de Mann-Whitney com correção de Bonferroni (n = 302).

Nove das 13 variáveis contextuais apresentaram associação significativa com o ICSP na análise bivariada (p < 0,001), conforme apresentado na Tabela 2 e na Figura 3. As variáveis que não apresentaram associação significativa foram: tipo de hospital (geral ou especializado), presença de leitos de UTI, leitos cirúrgicos e leitos de COVID-19.

**Tabela 2:** Estatística descritiva e análise da associação do Índice de Cultura de Segurança do Paciente (ICSP) e os fatores contextuais dos hospitais (n = 304).

Características	ICSP		
	Média	Mediana	Valor de p *
Região			< 0,001 **
Norte	52,5	51,6	
Nordeste	62,3	62,6	
Centro-oeste	58,0	59,3	
Sudeste	60,4	60,2	
Sul	56,3	57,2	
Tipo de estabelecimento			0,828 ***
Hospital geral	59,8	60,9	
Hospital especializado	60,4	60,2	
Porte do hospital			< 0,001 **
Pequeno (até 50 leitos)	66,0	68,9	
Médio (51-150 leitos)	62,0	62,4	
Grande (> 150 leitos)	56,9	57,3	
Leitos de UTI			0,915 ***
Não	59,9	59,7	
Sim	59,9	60,8	
Leitos cirúrgicos			0,724 ***
Não	61,4	60,5	
Sim	59,8	60,6	
Leitos de COVID-19			0,302 ***
Não	60,6	60,9	
Sim	59,3	60,4	
Hospital de ensino			< 0,001 ***
Não	62,9	63,9	
Sim	55,8	56,3	
Hospital público			< 0,001 ***
Não	64,4	64,8	
Sim	56,2	56,6	
Natureza jurídica			< 0,001 **
Administração pública	56,2	56,6	
Entidade empresarial	67,3	68,8	
Entidade filantrópica	62,4	62,3	
Gerência			< 0,001 **
Pública direta	56,8	57,1	
Pública indireta	56,2	56,8	
Privada filantrópica	62,6	62,9	
Privada empresarial	66,2	68,2	
Atende ao SUS			< 0,001 ***
Não	66,5	68,5	
Sim	58,6	59,4	
Leitos do SUS (%)			< 0,001 **
0	66,5	68,5	
1-49	65,1	67,6	
50-99	58,0	59,0	
100	58,1	58,5	
Público de fato (> 50% leitos do SUS)			< 0,001 ***
Não	66,1	68,2	
Sim	58,0	58,8	

SUS: Sistema Único de Saúde; UTI: unidade de terapia intensiva.

* Nível de significância. Valores em negrito indicam associações estatisticamente significativas (p < 0,05);

** Teste de Kruskal-Wallis;

*** Teste U de Mann-Whitney.

Nota: os pontos referem-se às medianas do ICSP das variáveis: região geográfica, tipo de gerência e porte hospitalar. Linhas azuis indicam diferenças estatisticamente significativas (p < 0,05); vermelhas, não significativas (p ≥ 0,05), conforme teste de Mann-Whitney com correção de Bonferroni.

A Tabela 2 apresenta os valores médios e medianas do ICSP segundo todas as 13 variáveis contextuais. Nove variáveis apresentaram associação estatisticamente significativa com o ICSP na análise bivariada. Destaca-se o maior escore médio entre hospitais de pequeno porte (66,0) em relação aos de grande porte (56,9), bem como entre hospitais com natureza jurídica empresarial (67,3) frente aos de administração pública (56,2). Também se observaram escores mais altos em hospitais com gerência privada empresarial (66,2) e menor média em hospitais com gerência pública indireta (56,2). Quanto à participação no SUS, hospitais que não atendem ao sistema público apresentaram média superior (66,5) à dos que atendem (58,6), padrão que se repete ao analisar o percentual de leitos SUS, com queda progressiva do ICSP conforme aumenta o percentual de leitos públicos. Hospitais com 0% de leitos SUS apresentaram média de 66,5, enquanto aqueles com 100% SUS tiveram média de 58,1. Da mesma forma, hospitais classificados como “público de fato” (mais de 50% dos leitos SUS) apresentaram média inferior (58,0) à dos demais (66,1).

Entre as regiões, a média mais elevada foi observada no Nordeste (62,3) e a mais baixa no Norte (52,5). Além disso, hospitais não vinculados ao ensino apresentaram escore médio mais alto (62,9) do que os de ensino (55,8).

Para as quatro variáveis sem associação estatisticamente significativa, as médias foram similares entre os grupos: por exemplo, hospitais gerais (59,8) e especializados (60,4); com e sem leitos de UTI (ambos com 59,9); com e sem leitos cirúrgicos (61,4 *vs.* 59,8); e com ou sem leitos de COVID-19 (60,6 *vs.* 59,3). Essas diferenças pequenas e estatisticamente não significativas sugerem que tais fatores, apesar de relevantes estruturalmente, podem ter influência limitada sobre a cultura de segurança no conjunto avaliado.

As variáveis significativas na análise bivariada foram incluídas no modelo de regressão linear múltipla. O modelo, estimado pelo método enter com reamostragem por *bootstrap*, foi estatisticamente significativo (ANOVA Z = 13,8; p < 0,001) e explicou 37,4% da variância do ICSP (R^2^ = 0,374). Permaneceram significativas no modelo final: região geográfica, tipo de gerência, porte hospitalar e hospital de ensino. A Tabela 3 apresenta os coeficientes e os IC95% BCa.

A equação preditiva foi:

(2) ICSP=58,67+(-1,21×Norte)+(7,30×Nordeste)+(1,18×Centro-oeste)+(3,69×Sudeste)+(-6,97×GerênciaPúblicaDireta)+(-10,31×GerênciaPúblicaIndireta)+(6,81×Pequenoporte)+(3,17×Médioporte)+(-2,28×HospitaldeEnsino)

**Tabela 3:** Modelo preditivo do Índice de Cultura de Segurança do Paciente (ICSP) a partir das variáveis contextuais (n = 302).

Modelo (Método Enter; p < 0,001; R^2^ = 0,374)	β	IC95% BCa		Valor de p *
		Inferior	Superior	
(Constante)	58,67	55,74	61,67	< 0,001
Região = Norte	-1,21	-5,85	3,13	0,612
Região = Nordeste	7,30	4,77	9,71	< 0,001
Região = Centro-oeste	1,18	-3,12	5,28	0,601
Região = Sudeste	3,69	0,60	6,59	0,019
Gerência = Pública direta	-6,97	-9,44	-4,32	< 0,001
Gerência = Pública indireta	-10,31	-12,76	-7,83	< 0,001
Porte = pequeno (até 50 leitos)	6,81	3,05	10,61	0,001
Porte = médio (51-150 leitos)	3,17	0,98	5,51	0,008
Hospital de ensino	-2,28	-4,47	0,06	0,055

β: coeficiente da regressão linear múltipla; IC95% BCa: intervalo de 95% de confiança com correção *bootstrap* do tipo corrigido e acelerado pelo viés.

* Nível de significância. Valores em negrito indicam associações estatisticamente significativas (p < 0,05).

Nota: referência para as variáveis categóricas: Região Sul, gerência privada empresarial e hospital não universitário.

### Perfis preditivos extremos

Para um hospital com perfil mais favorável - Região Nordeste, gerência privada filantrópica ou empresarial, pequeno porte e sem atividade de ensino -, o ICSP previsto foi de 72,78. Já para um hospital com o perfil menos favorável - Região Norte, gerência pública indireta, grande porte e hospital de ensino -, o ICSP previsto foi de 44,87. A diferença de 27,91 pontos percentuais entre os extremos destaca a magnitude da influência dos fatores contextuais sobre a cultura de segurança nos hospitais brasileiros. Esses achados oferecem subsídios importantes para intervenções diferenciadas conforme o perfil institucional.

## Discussão

### Resumo dos principais achados

Este estudo analisou dados de mais de 42 mil profissionais em 304 hospitais brasileiros, revelando variações relevantes entre as dimensões da cultura de segurança. “Percepção de segurança” e “Expectativas e ações da direção/supervisão da unidade/serviço que favorecem a segurança” se destacaram positivamente, enquanto “Resposta não punitiva aos erros” teve os menores escores. O modelo preditivo explicou 37% da variabilidade do ICSP, com associações significativas com região geográfica, tipo de gerência, porte hospitalar e *status* de hospital de ensino. Hospitais do Nordeste, de pequeno porte, privados, com baixa vinculação ao SUS e com natureza jurídica empresarial apresentaram melhores escores na análise bivariada. Entretanto, após ajuste multivariado, permaneceram associadas ao ICSP apenas as variáveis região, tipo de gerência, porte hospitalar e hospital de ensino.

### Interpretação dos achados à luz da literatura

Os resultados se alinham a padrões internacionais documentados. Revisão sistemática com hospitais de 21 países identificou como fortalezas o trabalho em equipe dentro das unidades e a aprendizagem organizacional, e como fragilidades a resposta não punitiva aos erros, dimensionamento de pessoal, trabalho em equipe entre unidades e transições de cuidado ^15^. Na América Latina, metanálise recente confirmou esse padrão, mas evidenciando fragilidades na notificação de eventos e no apoio da liderança ^16^. Já em hospitais europeus, estudo comparativo conduzido em quatro países (Suécia, Espanha, Hungria e Croácia) identificou que, embora algumas dimensões tenham sido avaliadas como “adequadas” no questionário, os dados qualitativos revelaram inconsistências com as práticas observadas, indicando um descompasso entre o discurso e a prática de segurança ^17^.

Neste estudo, “Percepção de segurança” e “Expectativas e ações da direção/supervisão da unidade/serviço que favorecem a segurança” obtiveram escores elevados, sugerindo valorização do tema nas unidades. Em contrapartida, “Resposta não punitiva aos erros” teve os piores resultados, indicando a persistência de uma cultura punitiva que pode inibir a notificação de incidentes. A dimensão “Apoio da gerência à segurança” apresentou grande variabilidade, refletindo diferentes níveis de engajamento institucional.

Os dados foram coletados em 2021, durante a pandemia de COVID-19, contexto que pode ter afetado negativamente a percepção dos profissionais, sobretudo nas dimensões ligadas à liderança, clima de segurança e resposta a erros. Um estudo indica que a pandemia aumentou o estresse, reduziu a abertura à comunicação e reforçou práticas punitivas, enfraquecendo a cultura de segurança ^18^. No Brasil, já foi argumentado que uma cultura de segurança sólida favorece tanto a resposta a emergências quanto a qualidade assistencial em tempos normais, ao promover valores como aprendizado, trabalho em equipe e compromisso coletivo ^19^.

Assim, os achados devem ser compreendidos à luz desse cenário de excepcionalidade, sem perder de vista que a pandemia também criou oportunidades para aprendizado institucional e fortalecimento da cultura de segurança no SUS e nos serviços de saúde em geral. Dessa forma, esse estudo reforça a importância de que avaliações sejam acompanhadas de ações concretas que envolvam lideranças, estimulem o aprendizado com erros e sustentem práticas organizacionais mesmo em contextos adversos ^15^ ^,^ ^16^ ^,^ ^17^ ^,^ ^18^ ^,^ ^20^.

A cultura de segurança do paciente reflete valores, atitudes e práticas institucionais influenciadas por fatores estruturais (como tipo de gestão, porte e complexidade) e relacionais (como liderança e engajamento das equipes) ^11^. Neste estudo, hospitais de pequeno porte apresentaram maiores escores de cultura de segurança, possivelmente por estruturas menos hierarquizadas e maior proximidade entre lideranças e trabalhadores, o que pode favorecer a comunicação e a confiança mútua. Estudo com 1.740 hospitais nos Estados Unidos identificou associação mais forte entre experiência do paciente e prevenção de eventos adversos em unidades menores, sugerindo que menor complexidade organizacional pode facilitar práticas seguras ^19^.

Entretanto, no Brasil, esses achados devem ser interpretados com cautela. Hospitais de pequeno porte financiados pelo SUS costumam operar com deficiências estruturais, como ausência de controle de infecções, escassez de profissionais para urgência e parto, e descumprimento de normas sanitárias mínimas ^21^. Embora representem a maioria das unidades no país, sua contribuição para a oferta de leitos é limitada e podem gerar riscos importantes. Ainda assim, seu papel é estratégico em regiões com pouca oferta assistencial, especialmente fora dos grandes centros urbanos ^22^.

Nossa amostra também difere da de estudos anteriores por incluir hospitais públicos e privados. Neste estudo, 44,7% da amostra era composta por hospitais privados, que mostraram desempenho superior aos públicos no modelo multivariado. Parte da associação positiva com hospitais de pequeno porte pode estar relacionada ao fato de muitos desses hospitais serem privados, com maior agilidade na gestão. Apesar do controle estatístico por variáveis contextuais, como tipo de gerência, a possível colinearidade entre porte e natureza jurídica exige cautela interpretativa e sugere necessidade de estudos que explorem melhor essas interações.

Além disso, o melhor desempenho dos pequenos pode refletir mais as dificuldades enfrentadas pelos grandes hospitais - como alta rotatividade, maior fragmentação e complexidade - do que uma cultura robusta nos pequenos. A disseminação de práticas seguras em instituições maiores é mais desafiadora e requer estruturas proporcionais e mecanismos de coordenação eficazes.

Também foi observada associação significativa entre melhores escores de cultura de segurança e hospitais com menor dependência do SUS, tanto em termos de percentual de leitos quanto de condição de “público de fato”. Esses resultados sugerem que a intensidade da vinculação ao SUS pode refletir diferenças estruturais e operacionais que influenciam negativamente a cultura organizacional. No entanto, tais variáveis não permaneceram significativas no modelo ajustado, possivelmente por estarem correlacionadas com outras dimensões institucionais, como tipo de gerência e natureza jurídica.

Hospitais de ensino apresentaram os menores escores de cultura de segurança. Inseridos em contextos de alta complexidade, com múltiplas missões (assistência, ensino e pesquisa), enfrentam dificuldades adicionais, como a presença constante de estudantes e profissionais em formação, que gera rotatividade e pode dificultar a padronização de práticas seguras e enfraquecer a cultura de notificação de eventos adversos ^23^. Um estudo brasileiro aponta que, mesmo com políticas específicas de reestruturação, como a contratualização, essas instituições ainda enfrentam dificuldades para incorporar metas de qualidade nas rotinas, pois mantêm práticas conservadoras e se integram efetivamente às redes de atenção ^24^.

No caso da Empresa Brasileira de Serviços Hospitalares (EBSERH), que administra muitos desses hospitais universitários, observa-se esforço estruturado para institucionalizar práticas de qualidade e segurança. A criação de núcleos de segurança do paciente, de gestão da qualidade, protocolos assistenciais e o fortalecimento da gestão da clínica têm sido acompanhados de melhorias nos indicadores operacionais, como aumento de internações, redução de permanência média e ampliação de pessoal ^25^. No entanto, os desafios de consolidar a cultura de segurança nesses contextos permanecem e os resultados negativos não devem ser lidos como falha da EBSERH, mas sim como reflexo da complexidade estrutural e das limitações do modelo de contratualização como instrumento de transformação cultural ^24^ ^,^ ^25^.

Entre os hospitais públicos de gestão indireta, as unidades geridas por organizações sociais (OS) também apresentam peculiaridades. A maior autonomia contratual, financeira e administrativa das OS permite mais flexibilidade na contratação de pessoal e aquisição de insumos, com ganhos de eficiência e cumprimento de metas ^26^. Contudo, a lógica contratual centrada em resultados nem sempre se traduz em melhorias na cultura de segurança. Estudos mostram que essas unidades mantêm estruturas hierárquicas rígidas, alta rotatividade e tensão entre eficiência e qualidade, o que pode comprometer a construção de um ambiente organizacional seguro ^27^.

Nossos achados também dialogam com estudos anteriores que compararam tipos de gestão. Enquanto Andrade et al. ^28^ apontaram melhor desempenho das unidades de administração indireta, este estudo encontrou o oposto: pior desempenho entre os hospitais de gestão indireta, reforçando a variabilidade institucional e a necessidade de estudos que analisem essas diferenças em maior profundidade.

Por fim, a associação positiva com a Região Nordeste chama atenção. Apesar de desafios estruturais históricos, os hospitais desta região apresentaram escores mais altos de cultura de segurança. O uso de ferramentas inovadoras, como o software de avaliação da cultura de segurança do paciente lançado no Rio Grande do Norte, pode ter contribuído para esses avanços. Adicionalmente, iniciativas regionais voltadas à formação de profissionais e ao fortalecimento institucional na área da qualidade em serviços de saúde parecem ter desempenhado papel relevante, com a capacitação de mais de 120 gestores da qualidade atuantes na Região Nordeste (dados institucionais) ^29^. A análise do Programa Nacional de Avaliação dos Serviços de Saúde (PNASS) 2015-2016 também revelou progressos importantes na região em termos de gestão e produção do cuidado ^30^, sugerindo que políticas regionais bem orientadas podem reduzir desigualdades e fortalecer a cultura de segurança mesmo em contextos adversos.

### Monitoramento da cultura de segurança: desafios regulatórios e o papel dos atores externos

A iniciativa de monitorar nacionalmente os indicadores de cultura de segurança do paciente é fundamental para atingir os objetivos estratégicos do PNSP e fortalecer a cultura de segurança hospitalar no Brasil. Contudo, conforme recomenda a OMS ^1^, esse papel não deve ser restrito à vigilância sanitária, mas compartilhado entre gestores do SUS (nas esferas federal, estadual e municipal), universidades, conselhos de classe e associações ou sociedades científicas, como a Associação Brasileira de Saúde Coletiva (Abrasco) e a Sociedade Brasileira para a Qualidade do Cuidado e Segurança do Paciente (Sobrasp), ampliando a governança sobre a segurança do paciente no sistema de saúde.

Apesar de seu papel fundamental para proteger a população contra riscos e problemas sanitários em serviços de saúde, a vigilância sanitária enfrenta importantes limitações regulatórias, estruturais e institucionais no Brasil. Um levantamento jornalístico apontou que, durante a pandemia de COVID-19, ao menos 188 hospitais operaram sem alvará sanitário, evidenciando fragilidades na fiscalização e no cumprimento da legislação vigente ^31^. A desigualdade na aplicação das normas entre os setores público e privado, a falta de infraestrutura e a baixa autonomia das vigilâncias locais agravam o problema. Além disso, desafios legais e institucionais limitam a atuação da vigilância sanitária como poder de polícia administrativa, dificultando a implementação de fiscalizações eficazes ^32^.

A regulação sanitária no Brasil sofre pressões políticas e econômicas, comprometendo sua independência ^32^. Essas limitações impactam diretamente a consolidação da cultura de segurança, reforçando a necessidade de fortalecer as vigilâncias locais por meio de investimentos em infraestrutura, qualificação técnica, marcos legais mais robustos e mecanismos de coordenação interfederativa. Paralelamente, a adoção de políticas indutoras pode estimular uma regulação mais equitativa e eficaz, favorecendo a institucionalização da segurança do paciente como política pública transversal e estruturante no SUS.

### Forças do estudo

Este estudo representa uma contribuição relevante para o avanço da segurança do paciente no Brasil. Trata-se da primeira análise nacional em larga escala sobre cultura de segurança do paciente com inclusão de hospitais públicos e privados, o que amplia a aplicabilidade dos achados e oferece uma visão abrangente sobre o tema no contexto brasileiro. A utilização de um instrumento internacionalmente validado e culturalmente adaptado ao país reforça a solidez metodológica. Ademais, a adoção de regressão linear múltipla com técnica de bootstrapping após seleção de variáveis aumentou a robustez estatística das estimativas, conferindo maior confiabilidade aos resultados apresentados ^14^.

### Limitações

Embora o estudo inclua uma amostra ampla e diversa, algumas limitações devem ser consideradas. O uso de dados secundários, como os do CNES, pode introduzir viés devido a possíveis inconsistências ou desatualizações. A baixa taxa de resposta em alguns hospitais também representa uma limitação, embora análises complementares tenham mostrado que sua inclusão fortaleceu o modelo preditivo.

Adicionalmente, o percentual de participação dos hospitais foi desigual entre as Unidades da Federação, com sub-representação de estados populosos como São Paulo e Rio de Janeiro. Esse padrão pode ter influenciado a comparação entre regiões geográficas, inclusive a associação positiva observada na Região Nordeste e o maior escore de cultura de segurança, o que deve ser interpretado com cautela.

O desenho transversal impede a inferência de relações causais, limitando as conclusões às associações observadas. Além disso, a explicação de 37% da variação no ICSP indica que fatores adicionais, como liderança, outros fatores estruturais e engajamento das equipes, também influenciam a cultura de segurança do paciente, mas não foram mensurados neste estudo. Essas limitações destacam a necessidade de estudos futuros que incorporem variáveis organizacionais mais amplas.

### Implicações práticas e para a saúde pública

Os achados deste estudo oferecem subsídios importantes para o aprimoramento das políticas públicas de segurança do paciente no Brasil. Hospitais públicos, tanto de administração direta quanto indireta, apresentaram os menores escores no índice de cultura de segurança quando comparados a hospitais privados, inclusive os de caráter filantrópico. Essa diferença evidencia fragilidades estruturais e de governança no setor público que demandam atenção urgente, incluindo o fortalecimento da liderança, a valorização dos profissionais e a institucionalização de práticas de segurança nos diferentes níveis de complexidade hospitalar.

A associação positiva entre cultura de segurança e hospitais de pequeno porte pode não refletir, necessariamente, uma cultura mais robusta nessas unidades, mas sim as maiores dificuldades enfrentadas por hospitais de grande porte - como alta rotatividade, maior complexidade e fragmentação de processos - em disseminar práticas seguras de forma integrada. Assim, estratégias específicas para grandes hospitais devem ser desenvolvidas, considerando sua escala e complexidade, com estruturas proporcionais e mecanismos efetivos de coordenação das ações de segurança.

Outro achado relevante foi o desempenho significativamente superior dos hospitais das regiões Nordeste e Sudeste, mesmo após controle estatístico por variáveis contextuais. Isso aponta para o potencial das políticas regionais e iniciativas locais bem estruturadas. O caso do Nordeste, em particular, desafia expectativas baseadas em desigualdades históricas e demonstra que investimentos em formação, inovação e articulação regional podem produzir avanços consistentes na cultura de segurança do paciente. Essas experiências devem ser analisadas e, quando possível, replicadas em outras regiões do país.

Por fim, os resultados reforçam a necessidade de fortalecer a atuação das vigilâncias sanitárias locais, promovendo maior autonomia, capacitação técnica e coerência regulatória. Também é fundamental revisar os marcos legais e ampliar políticas indutoras que assegurem uma regulação mais equitativa e eficaz, garantindo que os avanços na cultura de segurança se tornem política pública estruturante em todo o sistema de saúde.

### Recomendações para pesquisas futuras

Estudos futuros devem aprofundar a investigação sobre a interação entre variáveis contextuais e organizacionais, como liderança, clima organizacional e engajamento das equipes. Abordagens longitudinais são essenciais para identificar relações causais e avaliar o impacto de intervenções ao longo do tempo. Análises qualitativas, inclusive com análises etnográficas, também podem oferecer insights mais profundos sobre barreiras e facilitadores específicos de diferentes tipos de hospitais. Além disso, integrar dados operacionais (como indicadores assistenciais e de desempenho institucional) com dados culturais pode ampliar a compreensão do papel do contexto organizacional na consolidação de práticas seguras e orientar a formulação de políticas mais efetivas e adaptadas à realidade dos serviços de saúde no Brasil.

## Data Availability

Os dados de pesquisa estão disponíveis mediante solicitação ao autor de correspondência.
